# Editorial: The Role of Sirtuin-1 in Cardiovascular and Renal Pathophysiology

**DOI:** 10.3389/fphys.2021.770386

**Published:** 2021-10-22

**Authors:** Francisca Rodriguez, Francesca Seta

**Affiliations:** ^1^Department of Physiology, University of Murcia and Biomedical Research Institute in Murcia (IMIB), Murcia, Spain; ^2^Vascular Biology Section, Boston University School of Medicine, Boston, MA, United States

**Keywords:** sirtuin-1 (SIRT1), cardiovascular disease, metabolic and oxidative stress, renal disease, inflammation

Silent information regulator factor 2-related enzyme 1 (SIRT1) is a nicotinamide adenine dinucleotide-dependent deacetylase, which can deacetylate histone and non-histone proteins and other transcription factors, leading to the regulation of a plethora of physiological functions. The catalytic activity of SIRT1 depends on the availability of cellular NAD+, therefore, it is intrinsically linked to the energy status of the cell, i.e., NAD+/NADH ratio. In energy excess conditions, such as high-fat diets, SIRT1 activity decreases because of decreased NAD+/NADH ratio, whereas a low-energy status such as fasting, calorie restriction, nutrient deprivation and exercise, could increase SIRT1 activity by increasing the NAD+/NADH ratio. Therefore, SIRT1 is an intracellular energy sensor which detects the concentration of intracellular NAD+, and uses this information to adapt cellular energy output to cellular energy requirements and the mammalian metabolic clock.

A further link between SIRT1 and metabolic and oxidative stress has been established in a variety of tissues, in which SIRT1 expression has been shown to boost the cellular anti-oxidant defense, in part via deacetylation and subsequent activation of the anti-oxidant transcription factor Nrf2 or mitochondrial protein p66shc. SIRT1 has also been found to regulate the activity and/or expression of peroxisome proliferator-activated receptor gamma coactivator 1-alpha, FOXO family, and p53, which are transcription factors known to regulate the activity of antioxidant enzymes, apoptosis, and cell growth. Overall, SIRT1 is a well-established functional link between metabolic activity and genome stability. As such, SIRT1 has been proposed as a viable therapeutic target against diabetes and other aging-associated diseases, in which metabolic and oxidative stresses are the major culprit, including cardiovascular and renal diseases, which are the focus of this Research Topic.

As reviewed in details in this Research Topic, SIRT1 modulates pathways that inhibit cell apoptosis, oxidative stress, autophagy, inflammation and fibrosis, and regulate lipid metabolism, blood pressure, cardiac function, and sodium balance. As throughly reviewed by Man et al., endothelial nitric oxide synthase (eNOS) is a major direct de-acetylation target for SIRT1, such that SIRT1 interacts with the calmodulin-binding domain of eNOS, enhancing eNOS activity and promoting endothelial NO-dependent vasoprotection. Conversily, NO is known to regulate SIRT1 expression establishing a positive eNOS/NO/SIRT1 feedback loop, not only in the endothelium but possibly also in perivascular fat, supporting vascular health during aging and diabetes/obesity. Interestingly, Lipphardt et al. provide novel insights into an aberrant secretome of SIRT1-deleted endothelial cells leading to the activation of pro-fibrotic tubulointerstitial myofibroblasts and associated renal disease, underscoring the importance of a unique paracrine signaling of endothelial cells on neighboring cells/tissues. In further support of a cross-talk among different cell types, Karbowska et al. report that levels of aortic SIRT1 and SIRT3 inversily correlate with platelet hyperactivity, and subsequent increased risk of thrombotic events in a rat model of uremic syndrome, suggesting that vascular SIRT1 and SIRT3 disregulation, may contribute to increased risk of developing atherothrombotic events in chronic kidney disease patients, a leading cause of death in this patient population.

Furthermore, SIRT1 has been shown to protect the arterial wall from atherosclerosis and maladaptive remodeling, in part, by suppressing nuclear factor (NF)-κB and transforming growth factor β (TGF-β) activation, as well as oxidant and extracellular matrix-degrading pathways. Consistent with these findings, we report that vascular smooth muscle SIRT1 is indespensible to preserve aortic wall structural integrity, in the settings of aortic aneurysm, as described in Budbazar et al.; therefore, we suggest that boosting SIRT1 activity and its potential synergy with the heme oxygenase-1 (HO-1) system, can become a valid therapeutic approach for vascular diseases characterized by chronic aortic wall remodeling.

Lastly, SIRT1 has been found to regulate cardiac function by deacetylating ion channels and mitochondrial proteins in cardiac myocytes, further supporting a crucial role of SIRT1 in cardiovascular homeostasis. In this regard, Favero et al. report that melatonin, an indoleamine with an excellent safety profile, improves cardiac function in a mouse model of obesity (*ob/ob* mice) by preserving SIRT1 activity, which closely correlated with its anti-oxidant and anti-inflammatory effects in cardiomyocytes. Similarly, in a small clinical trial with prediabetic obese individuals, Sardu et al. report that levels of SIRT1 in subcutaneous fat inversily correlates with circulating inflammatory and oxidative stress markers, as well as cardiac performance and intima-media thickness, suggesting that abdominal adipose SIRT1 could become a biomarker to stratify pre-diabetic vs. normo-glycemic obese individuals, and predict their cardiovascular risk.

In summary, we hope that this Research Topic provides the reader with an in depth overview on the latest research on SIRT1 biology, mechanisms of regulation, interactions with other signaling pathways, and among multiple cell types (endothelial cells, vascular smooth muscle cells, platelets, cardiomyocytes, fibroblasts and adipocytes), and on novel approaches to activate SIRT1, in particular, in the context of cardiovascular and renal pathophysiology. We would like to thank the authors for their excellent contributions, and their continuous efforts in understanding the multiple facets of this intriguing enzyme.

## Author Contributions

All authors listed have made a substantial, direct and intellectual contribution to the work, and approved it for publication.

## Conflict of Interest

The authors declare that the research was conducted in the absence of any commercial or financial relationships that could be construed as a potential conflict of interest.

## Publisher's Note

All claims expressed in this article are solely those of the authors and do not necessarily represent those of their affiliated organizations, or those of the publisher, the editors and the reviewers. Any product that may be evaluated in this article, or claim that may be made by its manufacturer, is not guaranteed or endorsed by the publisher.

